# Revision of Clinical Pre-Test Probability Scores in Hospitalized Patients with Pulmonary Embolism and SARS-CoV-2 Infection

**DOI:** 10.31083/j.rcm2401018

**Published:** 2023-01-10

**Authors:** Mijo Meter, Ognjen Barcot, Irena Jelicic, Ivana Gavran, Ivan Skopljanac, Mate Zvonimir Parcina, Kresimir Dolic, Mirela Pavicic Ivelja

**Affiliations:** ^1^Department of Cardiology, University Hospital of Split, 21000 Split, Croatia; ^2^Department of Surgery, University Hospital of Split, 21000 Split, Croatia; ^3^Department of Infectious Diseases, University Hospital of Split, 21000 Split, Croatia; ^4^Department of Pulmology, University Hospital of Split, 21000 Split, Croatia; ^5^Department of Radiology, University Hospital of Split, 21000 Split, Croatia; ^6^University Department of Health Studies, University of Split, 21000 Split, Croatia; ^7^School of Medicine, University of Split, 21000 Split, Croatia

**Keywords:** pulmonary embolism, SARS-CoV-2 infection, pre-test probability scores

## Abstract

**Background:**

The need for computed tomography pulmonary angiography (CTPA) to rule out pulmonary embolism (PE) is based on clinical scores in association with D-dimer measurements. PE is a recognized complication in patients with SARS-CoV-2 infection due to a pro-thrombotic state which may reduce the usefulness of preexisting pre-test probability scores.

**Aim:**

The purpose was to analyze new clinical and laboratory parameters while comparing existing and newly proposed scoring system for PE detection in hospitalized COVID-19 patients (HCP).

**Methods:**

We conducted a retrospective study of 270 consecutive HCPs who underwent CTPA due to suspected PE. The Modified Wells, Revised Geneva, Simplified Geneva, YEARS, 4-Level Pulmonary Embolism Clinical Probability Score (4PEPS), and PE rule-out criteria (PERC) scores were calculated and the area under the receiver operating characteristic curve (AuROC) was measured.

**Results:**

Overall incidence of PE among our study group of HCPs was 28.1%. The group of patients with PE had a significantly longer COVID-19 duration upon admission, at 10 vs 8 days, *p* = 0.006; higher D-dimer levels of 10.2 vs 5.3 μg/L, *p *< 0.001; and a larger proportion of underlying chronic kidney disease, at 16% vs 7%, *p* = 0.041. From already established scores, only 4PEPS and the modified Wells score reached statistical significance in detecting the difference between the HCP groups with or without PE. We proposed a new chronic kidney disease, D-dimers, 10 days of illness before admission (CDD-10) score consisting of the three aforementioned variables: C as chronic kidney disease (0.5 points if present), D as D-dimers (negative 1.5 points if normal, 2 points if over 10.0 μg/L), and D-10 as day-10 of illness carrying 2 points if lasting more than 10 days before admission or 1 point if longer than 8 days. The CDD-10 score ranged from –1.5 to 4.5 and had an AuROC of 0.672, *p *< 0.001 at cutoff value at 0.5 while 4PEPS score had an AuROC of 0.638 and Modified Wells score 0.611. The clinical probability of PE was low (0%) when the CDD-10 value was negative, moderate (24%) for CDD-10 ranging 0–2.5 and high (43%) when over 2.5.

**Conclusions:**

Better risk stratification is needed for HCPs who require CTPA for suspected PE. Our newly proposed CDD-10 score demonstrates the best accuracy in predicting PE in patients hospitalized for SARS-CoV-2 infection.

## 1. Introduction 

In late December 2019, a novel coronavirus, SARS-CoV-2, was isolated from patients with bilateral pneumonia. Soon after, the clinical syndrome caused by SARS-CoV-2 was labeled COVID-19 by the World Health Organization. This highly transmissible and virulent disease has had a devastating effect, overwhelming hospitals worldwide with critically ill patients. Although knowledge of the wide range of clinical features of COVID-19 is growing fast, the pathophysiology underlying the most common complications has not yet been fully elucidated [1, 2]. COVID-19 is a systemic disease associated with vascular inflammation and endothelial injury. The role of hypercoagulability is certainly significant in the diverse clinical manifestations of COVID-19 [3]. The emergence of thromboembolic complications is common, with thrombotic complexity and coagulation disorders emerging as a critical issue in COVID-19 patients who consequently sustain an increased risk of pulmonary thromboembolism (PE) [4]. While coagulation disorders often occur in severe cases with poor prognosis, the nature of this abnormality is not yet clear. Moreover, there is a lack of explicit indications regarding the best algorithm for diagnosing PE in COVID-19 patients. In particular, it is unclear whether the latest guidelines issued in 2019 by the European Respiratory Society and the European Society of Cardiology for the diagnosis and therapy of acute PE can be successfully applied to COVID-19 patients with clinical characteristics of PE [5, 6]. Prior to the COVID-19 pandemic, the Geneva and Wells scores were the most commonly employed to predict PE in the general population, either alone or in combination with D-dimer [7]. The combination of Wells and Geneva Scores, along with D-dimers, allows doctors to efficiently screen patients and minimize needless radiological imaging [8]. PE Rule-out criteria (PERC) and the YEARS clinical decision rule are used to identify individuals who are unlikely to have a PE [9, 10]. Another excellent pretest probability measure for ruling out PE and reducing imaging tests is the 4-Level Pulmonary Embolism Clinical Probability Score (4PEPS) [11]. However, because COVID-19 patients have a distinct thrombotic environment, the utility of these scores in predicting PE has not been thoroughly studied. Herein, we aimed to analyze new clinical and laboratory parameters and compare existing scoring systems (Modified Wells score, Revised Geneva Score, Simplified Geneva Score, PERC score, 4PEPS score, YEARS Score) used in assessing the clinical likelihood of PE in patients with SARS-CoV-2 infection.

## 2. Materials and Methods

### 2.1 Study Type and Setting

This was a single-center, retrospective cohort observational study performed at a tertiary hospital (University Hospital of Split, Croatia). The study included only hospitalized individuals who had a confirmed SARS-CoV-2 infection for whom CT pulmonary angiography (CTPA) was performed due to clinical suspicion of PE. We analyzed the records of patients hospitalized in the Internal Medicine ward between February 2020 and August 2021, with WHO diagnostic criteria for COVID-19 pneumonia. Inclusion criteria was the SARS-CoV-2 infection confirmed by RT-PCR from a nasopharyngeal swab [12]. Patients diagnosed with a deep venous thrombosis (DVT) prior to performance of CTPA were excluded from the study, as were patients directly transferred to the Intensive Care Unit (ICU) due to severe hemodynamic or respiratory instability and an inability to undergo immediate CTPA. Patients with negative RT-PCR upon admission were also excluded.

### 2.2 Data Extraction

CTPA was performed and recorded in the radiological database related to the patients with COVID-19 infection. After obtaining the imaging findings from this database, the data of the hospitalized patients were acquired. Patient demographics, comorbidities, treatment methods, clinical, and laboratory data were gathered from electronic medical records (upon admission).

Items of all prediction algorithms (Modified Wells score [13, 14], Revised and Simplified Geneva score [15, 16], YEARS algorithm [10], PERC rule-out criteria [9], 4PEPS score [11]) were retrospectively calculated from the electronic medical records by two independent investigators (M.P.I and O.B). For scores that are continuous variables, the mean value between the ratings was used in the analysis, while for dichotomous scores, the disagreement was resolved by a third rater (M.M).

### 2.3 Variables and Outcomes

The presence of risk indicators, such as chest discomfort, dyspnea, sinus tachycardia or apparently new right bundle branch block (RBBB) on the ECG, deterioration of oxygen saturation, and increased D-dimer levels (upon admission or its increase during further hospitalization) raised clinical suspicion of PE. Based on a patient’s clinical condition and laboratory values upon admission, if the PE was considered as a first diagnosis, CTPA was immediately performed. Given that patients suspected of having PE as the primary diagnosis were screened, CTPA was performed as soon as technical possibilities allowed, i.e., within the first 24 hours of hospitalization.

The main aim of this study was the identification of clinical or laboratory parameters that could improve the accuracy of the existing scoring systems for PE prediction during SARS-CoV-2 infection. The secondary aim was the comparison of the scores and validation in our SARS-CoV-2 cohort.

### 2.4 Acquisition Protocol

After an intravenous injection of 60 to 90 mL of iodinated contrast agent, CTPA was acquired using a 128 multi-slice CT detector (Philips, Ingenuity CT, Registration Number: 0343E2012 SSA, Phillips Medical Systems (Cleveland) INC., Cleveland, OH, USA). The diagnosis of PE was based on pulmonary artery filling abnormalities. Furthermore, PE was diagnosed as subsegmental, segmental, or lobar by a competent radiologist, as is standard practice in our clinic.

### 2.5 Bias

The most significant possible source of bias might be the incomplete outcome data due to the large percentage of the patients excluded from the study (missing all relevant data). Another source of bias is the selection of patients on whom the CTPA was conducted. Another bias might develop if the clinically unstable patient was quickly transported to the ICU, removing the potential of CTPA verification.

To reduce the possibility of bias when calculating prediction scores (WELLS, 4PEPS, YEARS), two independent raters calculated the scores.

### 2.6 Study Size

The study included a consecutive cohort of patients admitted with the diagnosis of COVID-19 pneumonia in the University Hospital of Split, Croatia, for whom CTPA was performed before the COVID-19 vaccines were widely available.

### 2.7 Statistical Analysis

Absolute and relative frequencies were used to present categorical data. The normality of the datasets was tested by the Shapiro-Wilk test. Continuous data were described by the median followed by the respective interquartile range (IQR). To compare the medians between two groups, the Mann-Whitney U test was used while analysis of the differences between proportions was analyzed with Fisher’s exact test. Logistic regression analysis (univariate, multivariate: stepwise method) was utilized to analyze independent predictors associated with the possibility of PE. According to multivariate analysis, a new CDD-10 (Chronic kidney disease, D-dimers, 10 days of illness before admission) scoring system was created based on the coefficients of the regression model (Tables 2,3). Stratification of the score result was performed according to the distribution of the variable values (Table 4, Ref. [11, 14]).

For continuous variables in the scoring systems, an Intraclass Correlation Coefficient (ICC) was used as a measure of the reliability between two independent ratings. The two-way random-effects model was used, while mean values of ratings were used for further analysis. The ICC was presented and graded as a measure of consistency between the raters. For dichotomous scores, the inter-rater agreement was presented as Cohen’s weighted kappa [17].

The receiver operating curve (ROC) was used to determine the optimal thresholds, the area under the curve (AUC), specificity, and sensitivity of the tested predictors. All *p* values were two-sided and the level of significance was set at 0.05. Statistical analysis was performed using MedCalc® Statistical Software version 19.6 (MedCalc Software Ltd, Ostend, Belgium; https://www.medcalc.org; 2020).

## 3. Results

**Fig. 1.**
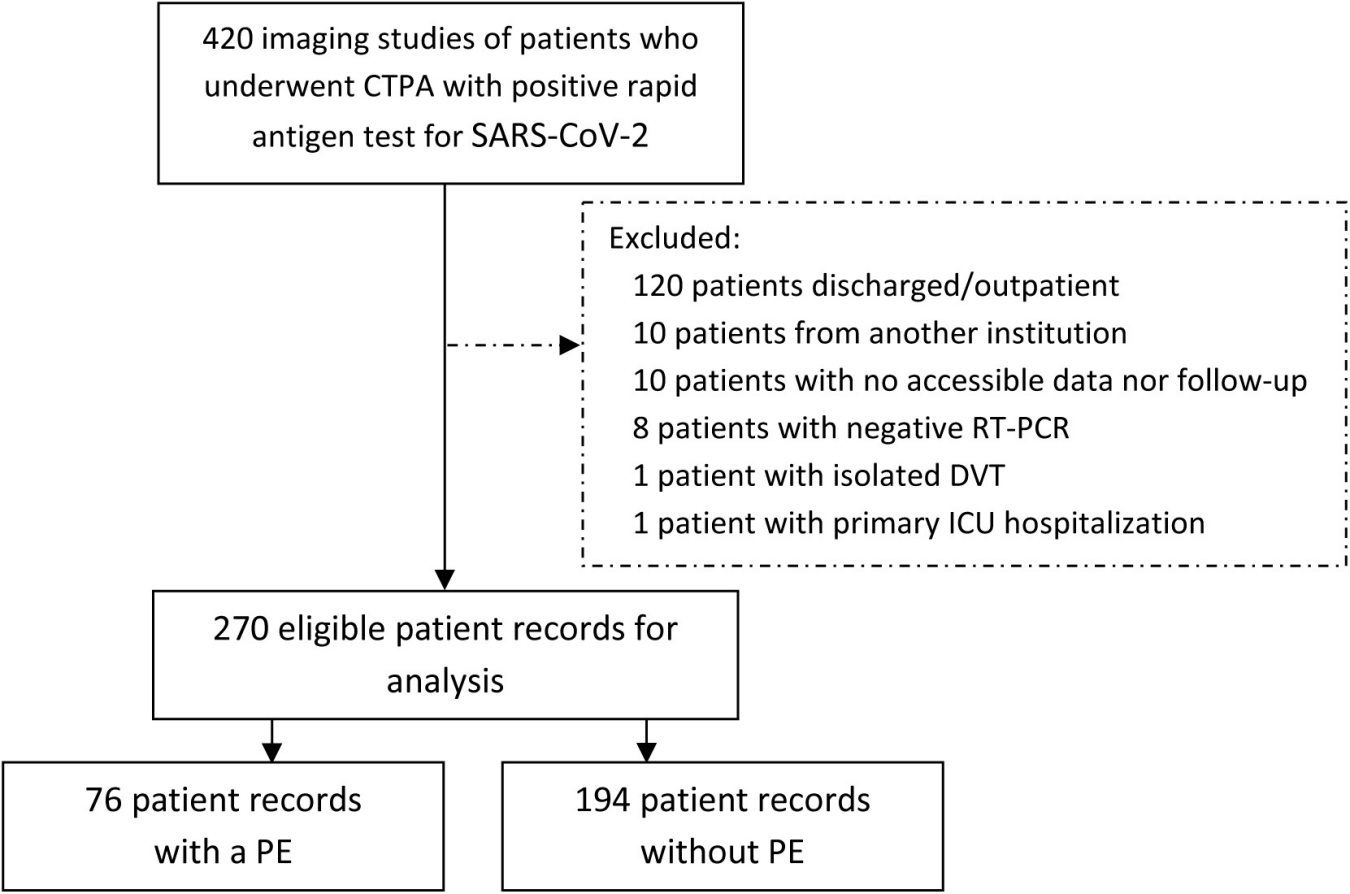
**Flowchart of the included patients who underwent CTPA due to clinical suspicion of PE**. CTPA, computed tomography pulmonary angiography; DVT, deep vein thrombosis; ICU, intensive care unit; PE, pulmonary embolism; RT-PCR, real-time reverse transcriptase-polymerase chain reaction.

From February 2020 to August 2021, 420 patients with dyspnea and positive rapid antigen test underwent CTPA due to suspected PE. 140 patients could not be found in the hospital information system: 120 were discharged immediately after CTPA was performed, 10 were sent back for scanning to a separate institution from which they were sent, and 10 had no records or were lost to follow-up. These patients, for whom none of the data relevant for this study was available, were excluded from the study. An additional eight patients were excluded due to negative RT-PCR test for SARS-CoV-2, while two others were excluded due to DVT and immediate admission to ICU (Fig. 1).

The characteristics of included patients and the differences in biometrics, comorbidity, biochemical parameters, and treatment outcomes according to PE are shown in Table 1. Overall, 76 out of 270 included patients had PE registered on CTPA. The incidence of PE in our cohort was 28.1% (95% CI: 21.8–34.5).

**Table 1.** **Difference in characteristics of patients according to PE**.

		PE (N = 76)	No PE (N = 194)	*p*-value*
Biometrics				
	Age [years]	70 (63–79)	69 (61–79)	0.456
	Female gender [n/N (%)]	57.9% (40.8–75.0)	60.1% (49.2–71.0)	0.833
	Cough [n/N (%)]	65.8% (47.6–84.0)	60.6% (49.6–71.6)	0.628
	Dyspnea [n/N (%)]	67.1% (48.7–85.5)	62.4% (51.3–73.5)	0.661
	Fever >37.3 °C [n/N (%)]	85.3% (64.4–106.2)	83.5% (70.6–96.4)	0.883
	Illness prior to admission [days]	10 (7–14)	8 (6–12)	0.006
	Heart rate [$\min^{-1}$]	90 (80–100)	90 (80–100)	0.828
	SpO2 [%]	90 (81–95)	91 (84–95)	0.220
Comorbidity, chronic therapy, habits				
	Prior Pulmonary Embolism [n/N (%)]	0.0% (0.0–0.0)	1.0% (–0.4–2.5)	0.375
	Prior DVT [n/N (%)]	5.3% (0.1–10.4)	3.6% (0.9–6.3)	0.545
	Prior thromboembolic event [n/N (%)]	5.3% (0.1–10.4)	4.1% (1.3–7.0)	0.690
	Prior stroke [n/N (%)]	9.2% (2.4–16.)	4.1% (1.3–7.0)	0.111
	Malignancy [n/N (%)]	4.0% (–0.5–8.4)	9.3% (5.0–13.6)	0.158
	Hematological disease [n/N (%)]	4.0% (–0.5–8.4)	5.2% (2.0–8.3)	0.684
	Autoimmune disease [n/N (%)]	5.3% (0.1–10.4)	4.1% (1.3–7.0)	0.690
	COPD [n/N (%)]	5.3% (0.1–10.4)	5.2% (2.0–8.3)	0.972
	Chronic atrial flutter [n/N (%)]	7.9% (1.6–14.2)	8.8% (4.6–12.9)	0.826
	Chronic kidney failure [n/N (%)]	15.8% (6.9–24.7)	7.2% (3.4–11.0)	0.041
	Dyslipidemia [n/N (%)]	19.7% (9.7–29.7)	12.9% (7.8–17.9)	0.189
	Diabetes [n/N (%)]	23.7% (12.7–34.6)	21.6% (15.1–28.2)	0.750
	Arterial hypertension [n/N (%)]	59.2% (41.9–76.5)	49.5% (39.6–59.4)	0.320
	Heart failure [n/N (%)]	27.6% (15.8–39.4)	15.5% (9.9–21.0)	0.039
	Sleep apnea [n/N (%)]	0.0% (0.0–0.0)	0.5% (–0.5–1.5)	0.531
	Anticoagulants [n/N (%)]	8.0% (1.6–14.4)	8.3% (4.3–12.4)	0.932
	ACE inhibitors [n/N (%)]	40.0% (25.7–54.3)	27.3% (20–34.7)	0.093
	Statins [n/N (%)]	13.5% (5.1–21.9)	11.5% (6.7–16.2)	0.665
	Smoking [n/N (%)]	6.6% (0.8–12.3)	6.2% (2.7–9.7)	0.916
Biochemical parameters				
	Creatinine [µmol/L]	86 (69–108)	84 (68–102)	0.543
	CRP [mg/L]	77.7 (38.3–138.4)	84.0 (46.2–159.0)	0.486
	D dimers [µg/L]	10.22 (3.70–27.44)	5.29 (1.29–11.95)	<0.001
	Hemoglobin [g/L]	135 (127–149)	138 (127–147)	0.814
	LDH [U/L]	389 (297–519)	361 (270 –482)	0.230
	Lymphocytes [%]	0.95 (0.71–1.32)	0.87 (0.59–1.24)	0.091
	Neutrophil to lymphocyte ratio	7.47 (5.073–10.750)	7.43 (4.705–12.405)	0.693
	Neutrophils [%]	7.57 (5.34–10.24)	6.56 (4.54–9.23)	0.059
	Platelets [${\mathrm{10}}^{9}$/L]	289 (187–362)	239 (171–321)	0.015
	Prothrombin time ratio	1.07 (0.89–1.25)	1.10 (0.94–1.21)	0.499
	hs-Troponin [ng/L]	22.2 (11.5–38.7)	13.1 (8.700–23.700)	0.006
	Leukocyte count [${\mathrm{10}}^{9}$/L]	9.4 (7.0–12.1)	8.2 (5.9–11.0)	0.030
	NT-proBNP [pg/mL]	538 (186–1948)	430 (192–1099)	0.453
Treatment/outcomes				
	NSAID therapy [n/N (%)]	0 (0–0)	0 (0–0)	0.721
	Antiviral therapy [n/N (%)]	13.3% (5.1–21.6)	19.3% (13.1–25.5)	0.299
	Steroid therapy [n/N (%)]	84.0% (63.3–104.7)	84.8% (71.8–97.9)	0.948
	LOS [days]	10 (6–15)	11 (5–17)	0.702
	NIV [n/N (%)]	1.3% (–1.3–3.9)	2.6% (0.3–4.9)	0.534
	HFNC [n/N (%)]	17.3% (7.9–26.8)	23.4% (16.6–30.3)	0.336
	ICU transfer [n/N (%)]	10.7% (3.3–18.1)	14.1% (8.8–19.4)	0.491
	Death [n/N (%)]	14.7% (6.0–23.3)	13.0% (7.9–18.1)	0.742

*For continuous variables Mann-Whitney U test, and for incidence rate comparison Chi-square test. 4PEPS, 4-Level Pulmonary Embolism Clinical Probability Score; COPD, Chronic Obstructive Respiratory Disease; DVT, deep venous thrombosis; HFNC, high-flow nasal cannula; ICU, intensive care unit; LOS, length of hospital stay; NIV, non-invasive ventilation; NSAID, non-steroid inflammatory drug; PE, pulmonary embolism; PERC, pulmonary embolism rule-out criteria; YEARS, YEARS study algorithm.

For those admitted due to respiratory difficulties, the group of patients with PE had a significantly longer period of the disease duration (10 days) compared to the patients with no PE (8 days) (*p* = 0.006). The D-dimer and hs-Troponin levels were significantly higher in the PE group (respective 10.2 μg/L vs 5.3 μg/L, *p *< 0.001; 22.2 ng/L vs 13.1 ng/L, *p* = 0.005). In the PE group, higher counts of platelets (289 vs 239 [×${\mathrm{10}}^{9}$/L], *p* = 0.016) and leukocytes (9.4 vs 8.2 [×${\mathrm{10}}^{9}$/L], *p* = 0.030) were observed. Also, the PE group contained more patients with CKD and heart failure (Table 1).

The consistency of averages of WELLS and 4PEPS scores calculations were both excellent: ICC of 91.2% (95% CI: 88.7–93.1) and 90.1% (95% CI: 93.3–95.9), respectively. The inter-rater agreement on YEARS score was very good: Cohen’s kappa = 82.9% (95% CI: 73.2–92.7).

### 3.1 The CDD-10 Score

**Fig. 2.**
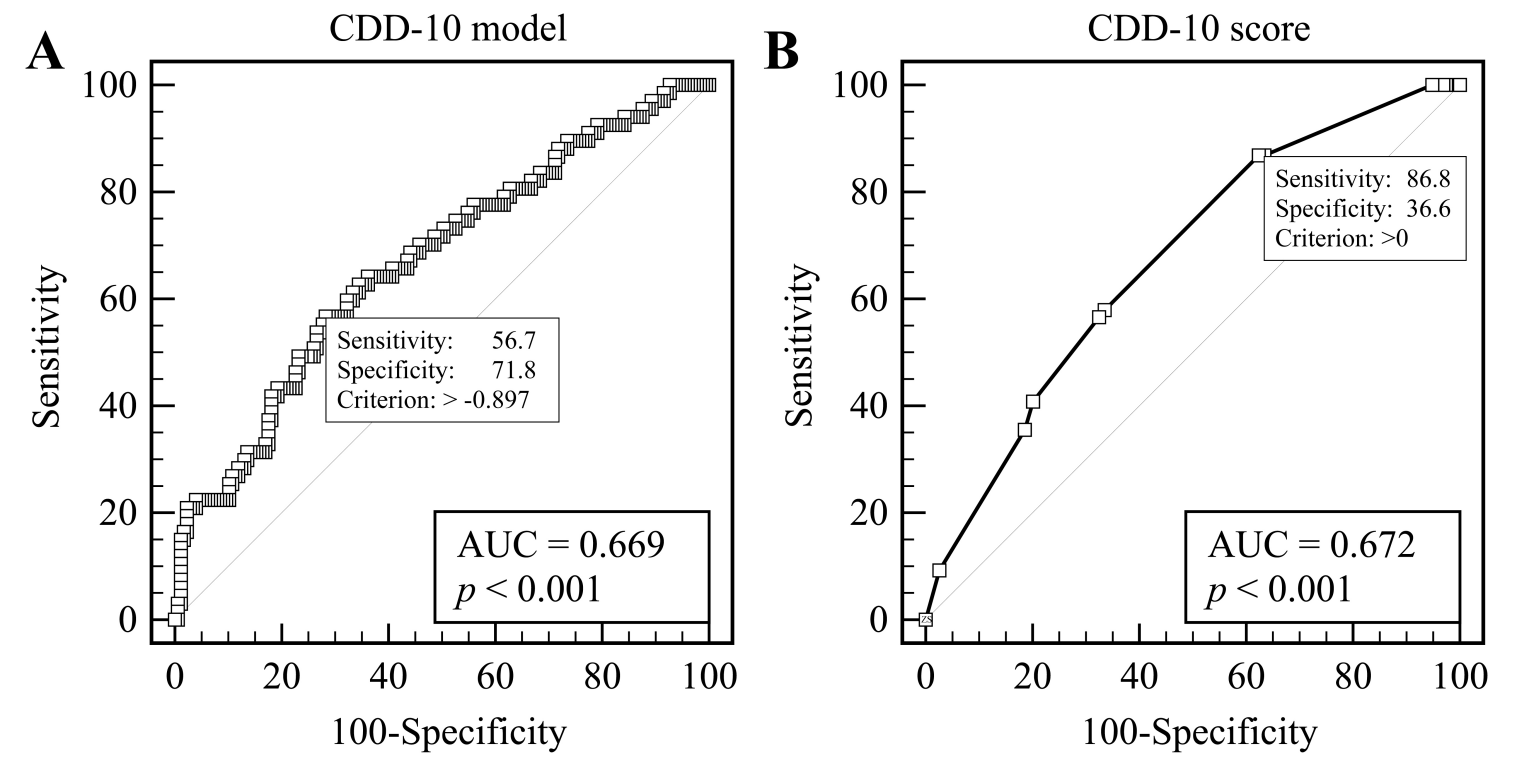
**Receiver operating curve analysis according to PE**. (A) Original CDD-10 regression model. (B) CDD-s10 scoring system.

The univariate logistic analysis associated eight possible predictors with the possibility of PE, including two existing scores: 4PEPS and modified Wells (**Supplementary Table 1**). Three of the independent predictors gave a unique statistically significant contribution to the multiple logistic regression model, namely, D-dimers, days of illness before admission, and the pre-existence of chronic kidney disease (χ^2^ = 21.04, DF = 3, *p *< 0.001). Based on this, a new proposed score consisting of three variables was defined: chronic kidney disease, D-dimers, and 10 days of illness before admission (CDD-10 score). This score accurately classifies 75.4% of PE cases, with cutoff value set at 0.5 resulting in AuROC 0.672, *p *< 0.001 (Tables 2,3, Fig. 2A).

**Table 2.** **Multiple logistic regression model coefficients and developed scoring system**.

Variable	Coefficient	Std. error	*p*	Odds ratio	Variable value	CDD-10 score points
D-dimer	0.0308	0.0123	0.013	1.03	<0.5 μg/L	–1.5
					>10.0 μg/L	2
					>20.0 μg/L	3
Illness duration prior to admission	0.0724	0.0284	0.011	1.08	>8 d	1
					>11 d	2
Existing chronic kidney disease	1.023	0.467	0.029	2.78	Yes	0.5
Constant	–2.193	0.367	<0.001			

**Table 3.** **Comparison of different instruments predicting pulmonary embolism**.

		PE (N = 76)	No PE (N = 194)	*p*-value*
Scores/Criteria for PE				
	4PEPS [pts]	5 (3–8)	4 (2–6)	<0.001
	Modified Wells [pts]	3.0 (0.8–3.3)	1.5 (0.0–3.0)	0.004
	Revised Geneva score [pts]	4 (4–6)	4 (3–6)	0.557
	Simplified Geneva score [pts]	2 (2–3)	2 (1–3)	0.354
	PERC (PE excluded) [n/N (%)]	1.3% (–1.3–3.9)	6.3% (2.7–9.9)	0.099
	YEARS (PE excluded) [n/N (%)]	7.9% (3.0–16.4)	14.4% (9.8–20.2)	0.160
Scores/models		AUC (95% CI)	Difference (95% CI) †	*p*-value
	CDD-10 model	0.69 (0.61–0.73)	0.00 (–0.04–0.05)	0.890
	CDD-10 score	0.67 (0.61–0.73)	/	/
	4PEPS	0.63 (0.56–0.69)	0.05 (–0.05–0.14)	0.329
	Modified Wells	0.59 (0.52–0.65)	0.084 (–0.01–0.18)	0.088

*For continuous variables Mann-Whitney U test, and for incidence rate comparison Chi-square test; † - compared to CDD-10 score.

The new scoring system proposes a low clinical probability (CP) of PE (less than 2%) at a CDD-10 score <0. A CDD-10 score of 0 to 2.5 corresponds to moderate CP (20–40%) and a CDD-10 score over 3 corresponds to a high CP (more than 50%). PE prevalence in the low category was 0%; moderate category, 23.9% (95% CI: 17.5%–31.7%); and in the high category, 42.9% (95% CI: 28.2%–62.4%), (Table 4, Fig. 2B).

**Table 4.** **Applicability and differences of the existing scores and CDD-10 scores by validation in different cohorts**.

	N	PE [n (%)]	Clinical Probability Score			
			Very low	Low	Moderate	High
4PEPS score			<0	0–5	6–12	>12
Original validation cohort #1 [11]	1548	332 (21.4%)	3 (2.5%)	76 (10.0%)	207 (34.1%)	46 (75.4%)
Original validation cohort #2 [11]	1669	196 (11.7%)	5 (1.4%)	79 (8.2%)	95 (28.4%)	17 (68.0%)
Our COVID-19 cohort	267	76 (28.5%)	1 (7.1%)	39 (24.3%)	33 (41.2%)	0 (0.0%)
Our cohort and D-dimer <10 µg/L	267	76 (28.5%)	0 (0%)	1 (0.6%)	2 (2.27%)	0 (0.0%)
	N	PE [n (%)]	Clinical Probability Score			
			Low	Moderate	High	
Modified Wells score			0–1.5	2–6	6.5–12.5	
Original cohort [14]	1239	217 (17.5%)	25 (3.4%)	112 (27.8%)	80 (78.4%)	
Our COVID-19 cohort	267	76 (28.5%)	36 (22.8%)	34 (36.2%)	6 (40.0%)	
Our cohort and D-dimer <0.01 × Age	267	76 (28.5%)	2 (1.3%)	0 (0%)	0 (0%)	
	N	PE [n (%)]	Clinical Probability Score			
			Low	Moderate	High	
CDD-10 score			<0	0–2.5	>3	
Our COVID-19 cohort	270	74 (27.4%)	0 (0.0%)	47 (23.9%)	27 (42.9%)	
Our cohort and D-dimer <0.01 × Age	270	74 (27.4%)	0 (0.0%)	2 (1.0%)	0 (0%)	

4PEPS, 4-Level Pulmonary Embolism Clinical Probability Score; PE, pulmonary embolism.

### 3.2 Applicability of the Existing Scores in COVID-19 Patients

Only two prediction scores, 4PEPS and the modified Wells score, reached statistical significance in the difference between the groups with or without PE (respective 5 vs 4 points, *p *< 0.001 and 3.0 vs 1.5 points, *p* = 0.006, Table 1). The univariate analysis also associated the same scores with the possibility of PE (Table 2, **Supplementary Table 1**), while other scores did not reach the threshold of statistical relevance.

The application of 4PEPS score on our cohort revealed its AUC of 0.64 (95% CI: 0.58–0.70), more than 10% less than in both of its own validation cohorts #1 and #2 – corresponding AUC 0.79 (95% CI: 0.76–0.82) and 0.78 (95% CI: 0.74–0.81), both *p *< 0.001, Table 3. In our cohort, the distribution of occurrences of PE differed significantly from its original validation cohort #1 (χ^2^ = 32.86, DF = 3, *p *< 0.001) and original validation cohort #2 (χ^2^ = 8.87, DF = 3, *p* = 0.031), as seen in Table 4.

The application of the modified Wells score on our cohort revealed an AUC of 0.61 (95% CI: 0.55–0.67), similar to the 4PEPS score. The distribution of occurrences of PE in our cohort differed significantly from its original validation cohort (χ^2^ = 51.37, DF = 2, *p *< 0.001, Table 4).

## 4. Discussion

While COVID causes a tendency to thromboembolic incidents, the pathophysiological mechanism that causes disorders of the coagulation system, as well as the diagnostic procedure that would enable efficient selection of patients with a high risk of PE, are still not completely clarified [18]. The main finding of our study is the newly proposed CDD-10 score, based only on three clinical-laboratory criteria (D-dimers, days of illness prior to admission, and the presence of chronic kidney disease) which demonstrated the highest accuracy in predicting PE in patients with SARS-CoV-2 infection. According to our study, pre-test probability scores that were frequently used for PE prediction in the general population showed low sensitivity and specificity for PE prediction in the SARS-CoV-2 cohort of patients. We found no difference regarding age, gender, comorbidities, other biochemical parameters, outcomes, or other pre-test probability scores between the two groups, except for chronic renal and heart failure, platelet and leukocyte levels, troponin levels, D-dimer levels, and days of illness before admission which were significantly higher in PE patients than in non-PE patients.

In our cohort, for whom CTPA was performed for clinical suspicion of thromboembolic complications, the incidence of PE was 28.1%, significantly higher than in the usual population of non-COVID patients who visit the emergency department due to dyspnea [18]. We believe there are two main explanations for this: (1) COVID-19 as a disease in which the chance of developing PE is increased, and (2) the selection of patients in whom the clinician performed CTPA because PE was the most likely differential diagnosis [19]. According to these findings, the real incidence of PE in all patients with confirmed SARS-CoV-2 infection is still unclear and probably underestimated.

The problem or the advantage of all pre-COVID emergency PE-related scoring systems is that they exclude the possibility of PE and thus avoid excessive use of CTPA. Any such scoring system is based on the exclusion of PE or reducing the number of false-negative predictions. Therefore, no system aims for a good sensitivity (or the resulting high positive predictive value). Secondly, the negative predictive values of these systems must be precise. In our case, where a high incidence reached over 28%, as opposed to validation cohorts of observed scoring systems [11, 14] of 12, 18 or a maximum of 21%, this value must be even more reliable.

Precisely due to the low sensitivity, no score can have symmetrical receiver operating curves with the surface under the curve trending to 100%. A statistically significant reduction in area under ROC for existing scores, in contrast to their validation cohorts, supports our thesis that in the COVID-19 population, the scores for patients who present for dyspnea (with a high incidence of PE) are not sufficient. Their specificity is lower, which can be seen from the increase in the incidence of PE (exceeding the agreed intervals) in low and moderate clinical prediction score groups. In our study, PE was more often observed in patients with CKD. Several processes can account for these observations. Because of elevated levels of procoagulant factors, reduced endogenous anticoagulants, and fibrinolytic activity in CKD patients, they are at risk of clot formation and thrombosis [20].

In contrast to our findings, an Italian multi-center retrospective investigation of 689 COVID-19 patients found that CKD was not predictive of PE incidence [21]. However, in a study by Inge H.Y Luu *et al*. [22] COVID-19 patients with positive CTPA more often had CKD than patients in whom CTPA was negative for PE (*p *< 0.03), confirming our results. In addition to the aforementioned molecular pathways, patients with CKD are more likely to have other documented risk factors for PE, such as congestive heart failure and immobility. In our cohort, more prevalent PE in patients with a history of CHF can be explained by decreased left ventricular systolic function, increased venous stasis, and chronic inflammation in the cardiovascular system [23]. Patients with PE experienced a longer delay from beginning of symptoms to hospitalization in the Fauvel *et al*. [24] research, in line with our findings.

TnI levels were observed to be considerably higher in the PE group. As a result of pulmonary vascular obstruction, right ventricular pressure may contribute to right ventricular dilatation and myocardial ischemia, resulting in a rise in Troponin I (TnI) levels [25]. Furthermore, PE induces a rise in tension in the right ventricle and pericardium, which can constrict the coronary arteries and cause partial myocardial ischemia and necrosis of myocardial cells, resulting in the release of TnI [26]. On the other hand, increased levels of troponin can also be seen in the setting of actual SARS-CoV-2 infection causing myocardial damage by non-ischemic myocardial processes, such as acute respiratory infection, sepsis, systemic inflammation, pulmonary thrombosis, cardiac adrenergic hyperstimulation during cytokine storm syndrome, and perhaps myocarditis. In a systematic review of four studies including 374 patients, cardiac TnI levels were considerably higher in those with severe COVID-19 infection compared to those with non-severe disease (OR 25.6, 95% CI: 6.8–44.5) [27]. Furthermore, TnI elevation in many patients may be exacerbated by concurrent renal failure, which was found to be more frequent in the PE group in our research. It can be concluded that the elevated levels of troponin in our PE patients are multifactorially caused [28].

Many patients with SARS-CoV-2 infection and respiratory failure appeared to have hypoxemia out of proportion to the impairment in lung compliance, which might be explained by pulmonary thrombosis, in some cases subclinical or radiologically unconfirmed [29]. In a study by Mirsadraee *et al*. [30], D-dimer levels did not discriminate between patients with and without PE in which screening CTPA was performed for patients with COVID-19 on admission to the ICU. This contradicts the results of our study but can be explained by the fact that these were patients in the ICU where the inflammatory and hypercoagulable component is particularly emphasized independently of PE existence [30]. Although a significant number of studies have confirmed the association of elevated D-dimer levels with PE in COVID patients, increased D-dimer levels alone cannot be used to confirm PE diagnosis [31, 32, 33, 34]. This is all the more the case because D-dimer values are increased even in COVID-19 individuals who do not have PE due to thromboinflammation or COVID-19-associated coagulopathy [35, 36].

Considering the above, the results of our research are not surprising. Namely, the levels of D-dimers were significantly higher in our PE group compared to the non-PE group. Due to the high levels of D-dimers in COVID-19 patients, even in the absence of PE, some authors recommended a higher D-dimer threshold to select patients appropriate for CTPA, based on the Youden index [29, 35, 36, 37, 38]. Clinical suspicion of PE in patients with COVID-19 pneumonia is often diminished because the signs and symptoms of COVID-19 pneumonia mimic those of PE which sometimes remain unrecognized: the clinical presentation of PE may overlap with that of COVID-19 pneumonia which may hinder the recognition of PE symptoms in patients who are already complaining of dyspnea. As a result, current estimates may significantly underestimate the real PE incidence in COVID-19, as revealed by autopsy investigations [39].

Kirsch *et al*. [40] verified the utility of the Wells score in predicting PE in a retrospective cohort of 64 hospitalized COVID-19 patients (HCP). In this study, a Wells score of 4 or above was strongly linked with PE development (*p* = 0.04). The AUC-ROC curve for the prediction of PE in HCPs, calculated for an optimal value of Wells score between 1 and 2, was 0.54, lower than in our cohort of patients with PE [40]. The study by Scardapane *et al*. [41] found no significant correlation between Wells score and PE in a cohort of 43 HCPs (median age 65 years, 51.16% males), as opposed to the Revised Geneva Score, which was significantly higher in PE patients than in non-PE patients (mean 4 + 2 vs 2 + 2, *p* = 0.01). In our study, the Revised Geneva score did not reach statistical significance in the difference between the groups with or without PE. Polo Fritz *et al*. [42] conducted a similar research, based on 41 HCPs (median age 71.7 years, 73% females) undergoing CTPA. The Wells score was found not clinically useful for predicting PE.

Although Wells score did not prove to be reliable in predicting PE in COVID patients, it has been widely used to predict PE in the general population, stratifying patients into three groups with low (1.3% prevalence), moderate (16.2% prevalence), and high risk (37.5% prevalence), according to their pre-test chance of developing PE [43]. The score had an AUC of the ROC calculated for predicting PE in the general population of 0.632 (95% CI: 0.574–0.691) [44] which was similar to our cohort of patients with PE and COVID-19 (AUC 0.61 (95% CI: 0.54–0.67)).

The fundamental disadvantage of the Wells score is the inclusion of a physician’s subjective judgment among factors, i.e., “PE is the most likely diagnosis”, as previously stated by Klok and colleagues [45]. Especially in COVID-19 management, physicians will most often suspect PE if patients present with hypoxemia and tachycardia, thus limiting the utility of this score in predicting PE. It has been observed that using the Wells score and D-dimer together improves the test’s sensitivity and specificity [46, 47]. Kampouri *et al*. [47] discovered that a Wells score >2 paired with a D-dimer value >3000 ng/L offered a highly specific prediction rule with a sensitivity of 57.1% and a specificity of 91.6% in a retrospective investigation of 443 HCPs (median age 68.68 years, 57.7% males). To date, no studies have been conducted to assess the accuracy of the 4PEPS score in predicting PE in the SARS-CoV-2 cohort of patients. The 4PEPS score has an AUC of 0.63 (95% CI: 0.57–0.69) in our cohort of patients with SARS-CoV-2 infection, indicating that it is neither specific nor sensitive for predicting PE in patients with SARS-CoV-2 infection.

Compared to the existing scores, our newly proposed CDD-10 score, based only on three clinical-laboratory criteria (D-dimers, days of illness before admission, and existing CKD), showed the highest accuracy in predicting PE, with its AUC-ROC of 0.669 and 0.672, respectively. None of the other four scores (Revised and Simplified Geneva score, PERC, YEARS) reached statistical significance in our cohort of patients; according to our study, they should not be routinely used for PE prediction in patients with SARS-CoV-2 infection. YEARS algorithm and PERC are used to rule out PE, reducing unnecessary CTPA. In a retrospective study of 93 COVID-19 patients with acute respiratory failure, Porfidia *et al*. [48] found no difference in terms of age, gender, and PERC between COVID-19 patients with positive CTPA and those with negative CTPA for PE, with the exception of D-dimer >1000 ng/mL and the indication to undergo CTPA based on YEARS algorithm. However, there were patients without confirmed PE who had D-dimer >1000 ng/mL and an indication to undergo CTPA based on the YEARS algorithm, indicating that the YEARS algorithm is not sufficiently specific.

In our study, there was no difference between the PE group and the non-PE group regarding the YEARS algorithm and PERC. Accordingly, these algorithms should not be routinely used in the stratification of patients who need to undergo CTPA for suspected PE. According to our findings, one factor that may have the greatest influence on inter-rater reliability during the calculation of pre-test probability scores is “PE as a first or equally likely diagnosis”, based primarily on the physician’s impression during admission after considering the patient’s medical history and clinical status. “PE as a first or equally likely diagnosis” was mostly considered if patients had unexplained dyspnea, higher than expected D-dimer levels, or low or rapid drop in oxygen saturation not explained by concurrent pneumonia or other possible factors.

### 4.1 Strengths

The CDD-10 score that we developed to assess the risk of PE is based on simple clinical and laboratory criteria: The usual cut-offs for D-dimers (0.5 μg/L) and its easy-to-remember extreme multiples (10 and 20 μg/L); more than a week of illness or more than 11 days of illness; and the existence of chronic renal failure. A negative score generally excludes the need for CTPA.

### 4.2 Limitations

This study was retrospective, and it was done in a single Clinical Medical Centre in Split, Croatia, with a relatively small number of patients. Not all patients admitted with suspicion of PE were proven by CTPA and thus did not enter this study. A large percentage of patients had to be excluded due to missing data. This study evaluates hospitalized patients with SARS-CoV-2 infection; as such, it does not apply to outpatients nor ICU inpatients with suspected PE, since these patients were not included in this study. Only patients with highest risk who underwent CTPA were included in the study which may explain the high incidence of PE compared to the other studies. Other PE prediction algorithms are designed to be used in patients with only suspected PE, many of whom are at low risk and are ruled out by D-dimer testing and most of whom do not undergo CTPA. Therefore, the CDD-10 score should only be used in hospitalized COVID-19 patients (HCP). Clinical pre-test probability (CPTP) tools were not calculated for all in-patients, but the comparison was made for PE within a high-risk sample. However, we compared the performance of the CDD-10 score to the performances of other scores on respective validation cohorts of other scoring systems.

## 5. Conclusions

According to our study, previously used pre-test probability scores for PE showed low sensitivity and specificity for PE prediction in the SARS-CoV-2 cohort of patients. Only the 4PEPS score and Modified Wells score reached statistical significance in hospitalized COVID-19 patients (HCP). Our newly proposed CDD-10 score, based only on three clinical and laboratory parameters, demonstrated the highest accuracy in PE prediction among HCP. Further prospective studies are necessary to determine other risk factors and to develop algorithms for better risk stratification of SARS-CoV-2 patients who require CTPA for suspected PE.

## Data Availability

The data sets used and/or analyzed during the current study are available from the corresponding author on reasonable request.

## Abbreviations

AUC, Area under the ROC curve; CHF, Chronic heart failure; CDD-10, Chronic kidney disease, D-dimers, 10 days of illness before admission; CKD, Chronic kidney disease; CI, Confidence interval; COPD, Chronic Obstructive Pulmonary Disease; COVID-19, Coronavirus disease 2019; CPTP, Clinical pre-test probability; CTPA, Computerized tomography pulmonary angiography; DVT, Deep venous thrombosis; HFNC, High-flow nasal cannula; HCP, Hospitalized COVID-19 patients; ICU, Intensive Care Unit; IQR, Interquartile range; LOS, length of hospital stay; LVEF, Left ventricular ejection fraction; MV, Mechanical ventilation; NIV, Non-invasive ventilation; NSAID, Non-steroid inflammatory drug; OR, Odds ratio; PCR, Polymerase chain reaction; PE, Pulmonary embolism; 4PEPS, 4-Level Pulmonary Embolism Clinical Probability Score; PERC, Pulmonary embolism rule-out criteria; RBBB, Right bundle branch block; ROC, Receiver operating curve; RT-PCR, Reverse-transcriptase polymerase chain reaction; TnI, Troponin I; TPA, Tissue plasminogen activator; VTE, Venous thromboembolism; YEARS, YEARS study algorithm; WHO, World Health Organization.

## Supplementary Material

Supplementary material associated with this article can be found, in the online version, at https://doi.org/10.31083/j.rcm2401018.
