# Supplementary material for: Revision of Clinical Pre-Test Probability Scores in Hospitalized Patients with Pulmonary Embolism and SARS-CoV-2 Infection
Source: Rev Cardiovasc Med. 2023 Jan 10;24(1):18. doi: 10.31083/j.rcm2401018 (PMC11270401; doi:10.31083/j.rcm2401018)
Supplement: Supplementary file 1 [file 2153-8174-24-1-018-s1.zip › Supplementary TABLE 1.docx]

**Table 1 –** Univariate analysis of the possible predictors of pulmonary embolism with respective significance levels (*p*) and coefficients

| **Variable** | **Chi-squared** | **DF** | ***p*** | **Coefficient** | **Std. Error** | **Wald** | ***p*** | **Odds ratio** | **95% CI** |
| --- | --- | --- | --- | --- | --- | --- | --- | --- | --- |
| 4PEPS [pts] | 12.527 | 1 | 0.0004 | 0.157 | 0.046 | 11.917 | 0.0006 | 1.1701 | 1.0703 to 1.2793 |
| D dimers [µg/L] | 9.825 | 1 | 0.0017 | 0.037 | 0.012 | 9.910 | 0.0016 | 1.0373 | 1.0139 to 1.0612 |
| Illness prior to admission [days] | 8.842 | 1 | 0.0029 | 0.078 | 0.027 | 8.494 | 0.0036 | 1.0806 | 1.0257 to 1.1384 |
| Modified Wells [pts] | 7.850 | 1 | 0.0051 | 0.195 | 0.070 | 7.795 | 0.0052 | 1.2152 | 1.0598 to 1.3935 |
| Heart failure [n/N (%)] | 4.992 | 1 | 0.0255 | 0.736 | 0.324 | 5.146 | 0.0233 | 2.0873 | 1.1052 to 3.9419 |
| Leukocyte count [10^9/L] | 4.736 | 1 | 0.0295 | 0.051 | 0.030 | 2.881 | 0.0896 | 1.0525 | 0.9921 to 1.1165 |
| ACE inhibitors in chronic therapy [n/N (%)] | 6.515 | 1 | 0.0385 | 0.573 | 0.286 | 4.028 | 0.0448 | 1.7736 | 1.0135 to 3.1037 |
| Chronic kidney failure [n/N (%)] | 4.241 | 1 | 0.0395 | 0.880 | 0.419 | 4.401 | 0.0359 | 2.4107 | 1.0595 to 5.4852 |
| Platelets [10^9/L] |  |  | 0.0524 |  |  |  |  |  |  |
| PERC (PE excluded) [n/N (%)] |  |  | 0.0579 |  |  |  |  |  |  |
| SpO2 [%] |  |  | 0.1004 |  |  |  |  |  |  |
| Prior stroke [n/N (%)] |  |  | 0.1168 |  |  |  |  |  |  |
| Malignancy [n/N (%)] |  |  | 0.1176 |  |  |  |  |  |  |
| YEARS (PE excluded) [n/N (%)] |  |  | 0.1294 |  |  |  |  |  |  |
| Arterial hypertension [n/N (%)] |  |  | 0.1492 |  |  |  |  |  |  |
| Dyslipidemia [n/N (%)] |  |  | 0.1639 |  |  |  |  |  |  |
| hs-Troponin [ng/L] |  |  | 0.2394 |  |  |  |  |  |  |
| Antiviral therapy [n/N (%)] |  |  | 0.2416 |  |  |  |  |  |  |
| Prior Pulmonary Embolism [n/N (%)] |  |  | 0.2481 |  |  |  |  |  |  |
| LDH [U/L] |  |  | 0.2554 |  |  |  |  |  |  |
| HFNC [n/N (%)] |  |  | 0.2689 |  |  |  |  |  |  |
| CRP [mg/L] |  |  | 0.2793 |  |  |  |  |  |  |
| Prothrombin time ratio |  |  | 0.2876 |  |  |  |  |  |  |
| Sleep apnea [n/N (%)] |  |  | 0.4156 |  |  |  |  |  |  |
| Revised Geneva score [pts] |  |  | 0.4168 |  |  |  |  |  |  |
| Cough [n/N (%)] |  |  | 0.4296 |  |  |  |  |  |  |
| Neutrophil to lymphocyte ratio |  |  | 0.4445 |  |  |  |  |  |  |
| LOS [days] |  |  | 0.4525 |  |  |  |  |  |  |
| Simplified Geneva score [pts] |  |  | 0.4542 |  |  |  |  |  |  |
| Dyspnea [n/N (%)] |  |  | 0.4649 |  |  |  |  |  |  |
| Age [years] |  |  | 0.4874 |  |  |  |  |  |  |
| NIV [n/N (%)] |  |  | 0.5075 |  |  |  |  |  |  |
| Prior DVT [n/N (%)] |  |  | 0.5461 |  |  |  |  |  |  |
| Creatinine [µmol/L] |  |  | 0.5647 |  |  |  |  |  |  |
| Hemoglobin [g/L] |  |  | 0.5968 |  |  |  |  |  |  |
| Statins in chronic therapy [n/N (%)] |  |  | 0.6477 |  |  |  |  |  |  |
| Hematological disease [n/N (%)] |  |  | 0.671 |  |  |  |  |  |  |
| Prior thromboembolic event [n/N (%)] |  |  | 0.6877 |  |  |  |  |  |  |
| Autoimmune disease [n/N (%)] |  |  | 0.6877 |  |  |  |  |  |  |
| Fever >37.3°C [n/N (%)] |  |  | 0.7117 |  |  |  |  |  |  |
| Diabetes [n/N (%)] |  |  | 0.7188 |  |  |  |  |  |  |
| NSAID therapy [n/N (%)] |  |  | 0.7202 |  |  |  |  |  |  |
| Female gender [n/N (%)] |  |  | 0.7437 |  |  |  |  |  |  |
| NT-proBNP [pg/mL] |  |  | 0.7716 |  |  |  |  |  |  |
| Neutrophils [%] |  |  | 0.7987 |  |  |  |  |  |  |
| Chronic atrial flutter [n/N (%)] |  |  | 0.817 |  |  |  |  |  |  |
| Heart rate [min-1] |  |  | 0.8605 |  |  |  |  |  |  |
| Steroid therapy [n/N (%)] |  |  | 0.8686 |  |  |  |  |  |  |
| Smoking [n/N (%)] |  |  | 0.913 |  |  |  |  |  |  |
| Anticoagulants in chronic therapy [n/N (%)] |  |  | 0.9289 |  |  |  |  |  |  |
| Lymphocytes [%] |  |  | 0.9375 |  |  |  |  |  |  |
| COPD [n/N (%)] |  |  | 0.9712 |  |  |  |  |  |  |
